# Artificial intelligence in neuro-oncology

**DOI:** 10.3389/fnins.2023.1217629

**Published:** 2023-12-14

**Authors:** Vihang Nakhate, L. Nicolas Gonzalez Castro

**Affiliations:** ^1^Department of Neurology, Brigham and Women’s Hospital, Harvard Medical School, Boston, MA, United States; ^2^Massachusetts General Hospital, Harvard Medical School, Boston, MA, United States; ^3^Harvard Medical School, Boston, MA, United States; ^4^The Center for Neuro-Oncology, Dana–Farber Cancer Institute, Boston, MA, United States

**Keywords:** neuro-oncology, Artificial intelligence, brain tumor, glioma, glioblastoma, IDH gliomas mut

## Abstract

Artificial intelligence (AI) describes the application of computer algorithms to the solution of problems that have traditionally required human intelligence. Although formal work in AI has been slowly advancing for almost 70 years, developments in the last decade, and particularly in the last year, have led to an explosion of AI applications in multiple fields. Neuro-oncology has not escaped this trend. Given the expected integration of AI-based methods to neuro-oncology practice over the coming years, we set to provide an overview of existing technologies as they are applied to the neuropathology and neuroradiology of brain tumors. We highlight current benefits and limitations of these technologies and offer recommendations on how to appraise novel AI-tools as they undergo consideration for integration into clinical workflows.

## Introduction

**Figure 1:**
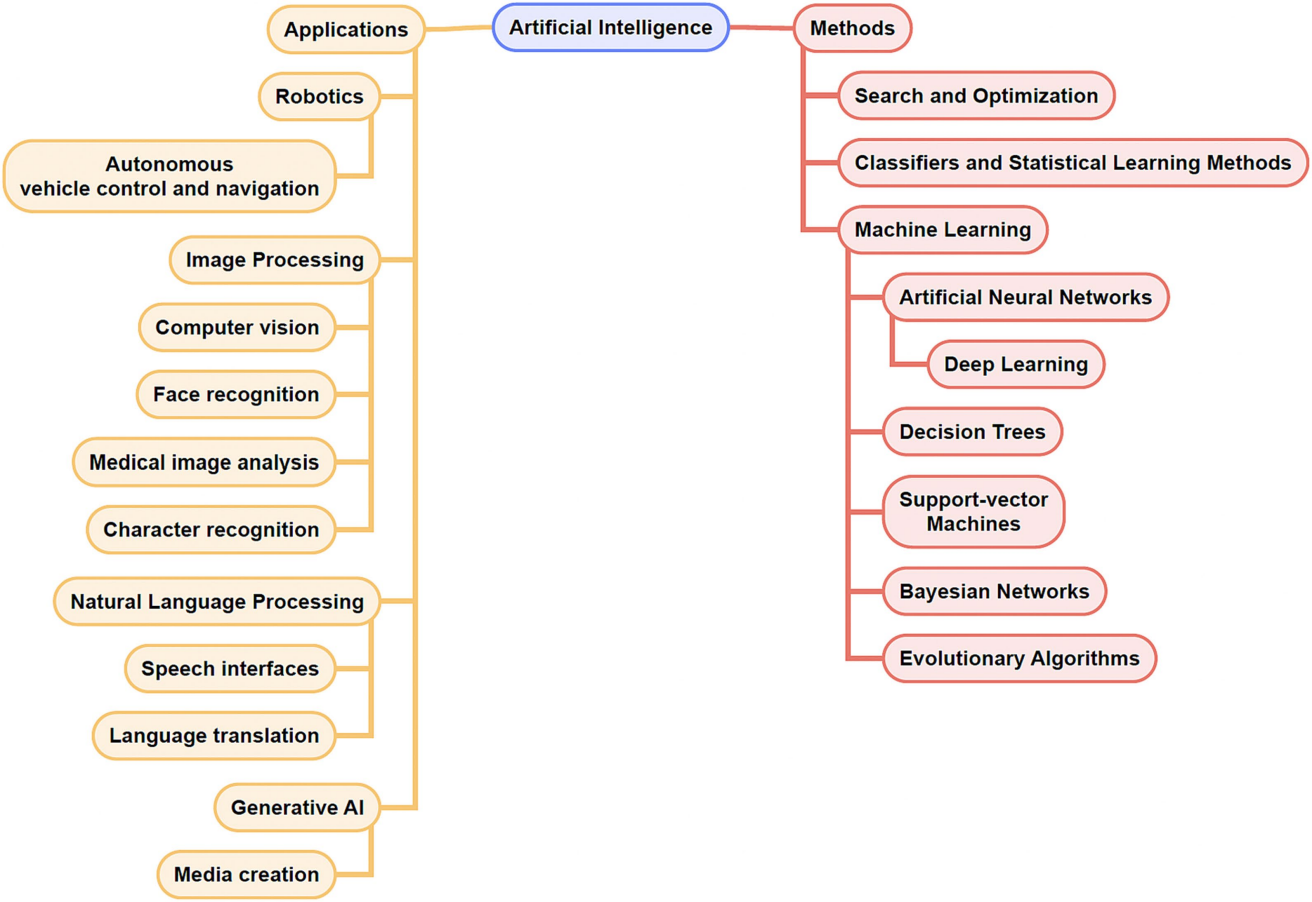
Concept map of select artificial intelligence (AI) applications and methods. Note that there exists overlap among some of the applications and methods listed.

As Artificial intelligence (AI) continues to shape and reshape various aspects of our physical and virtual lives, its growing impact on and promise in medicine are hard to ignore. One of the first definitions of “artificial intelligence” was formulated in 1956 by Prof. John McCarthy at Dartmouth University, to refer to “making a machine behave in ways that would be called intelligent if a human were so behaving.” (Nillson, 2010). In a broad sense, AI signifies machines that can simulate human intelligence with tasks like learning, visual processing, problem-solving, decision-making, and that increasingly can extend the reaches of human intelligence with enhanced classification and prediction. While Artificial General Intelligence (AGI), or “strong AI,” refers to systems that can perform a wide range of tasks comparably to humans, most existing systems are considered Artificial Narrow Intelligence (ANI), or “weak AI,” signifying systems capable of performing a defined task (Russel and Norvig, 2020). Narrow AI systems can be further classified based on physical (robotic/automation systems) and cognitive applications (machine learning, computer vision, natural language processing). Most AI applications in medicine are comprised of machine learning (ML) applications. ML refers to the ability of algorithms (see Table 1) to derive patterns and rules (“learn”) from large sets of data to recognize patterns, perform tasks or make predictions without being explicitly programmed to do so (Kann et al., 2021). Within ML, learning algorithms can be characterized as supervised (using data with labeled input–output pairs), unsupervised (using data without labeled inputs) or reinforcement (using a reinforcement feedback signal for learning). While conventional ML requires manual engineering of raw data to create representations suitable for ML algorithms to learn, deep learning (DL) refers to a subset of ML techniques that can extract and learn features from raw, unstructured and multimodal data (e.g., raw imaging, text, audio-visual data) using layered neural networks (LeCun et al., 2015). DL algorithms can be supervised or unsupervised (see Figure 1).

**Table 1:** Glossary of commonly used artificial intelligence terms.

Artificial intelligence (AI)	Computer algorithms that can solve problems, inform decision-making and perform complex tasks that have traditionally required human intelligence
Machine learning (ML)	Discipline in AI involving computers or “machines” as agents that can learn patterns and rules from large sets of data to build predictive models and solve problems, without being explicitly programmed to do so
Algorithm	Set of rules that an agent, in this case a “machine,” can follow to complete a set of tasks
Supervised learning	Machine learning in which the agent observes data consisting of input–output pairs with manually assigned labels, and learns a function that predicts output from input
Unsupervised learning	Machine learning in which the agent learns patterns in the input without any explicit manual feedback
Random forest algorithm	Type of supervised learning algorithm used for classification and regression tasks, in which a large number of decision trees operate together as an ensemble to reach a common output result
Support vector machine	Type of supervised learning algorithm used for classification and regression tasks, in which a subset of training points (support vectors) from the decision function are used to complete a given task
Deep learning (DL)	Machine learning techniques in which the computational path between input and output consists of multiple layers of simple, adjustable computing elements. The resulting computational circuit, termed a**neural network**, allows for a large number of input variables to interact in complex ways. Widely used in visual object recognition, speech recognition, image and speech synthesis.
Convolutional neural networks	Type of DL neural network architecture commonly used in analysis of images
Transfer learning	Machine learning method in which knowledge gained by an agent from one domain can be transferred and applied to a new domain, so that learning can proceed faster with less data
Large language model	A neural network-based model trained on large text datasets using self-supervised learning.

Adapted from *Artificial Intelligence: A Modern Approach*, Russel and Norvig (2020).

With the advent of increasing computing power in recent decades, DL has achieved remarkable results in areas including image classification, speech recognition, and game playing, among others (LeCun et al., 2015; Silver et al., 2016). Remarkable flexibility of input and output structures coupled with modern computing power have positioned ML and DL well to analyze large data sets that are increasingly being generated in modern medicine and oncology, and to aid in using such data to guide decision making.

**Figure 2:**
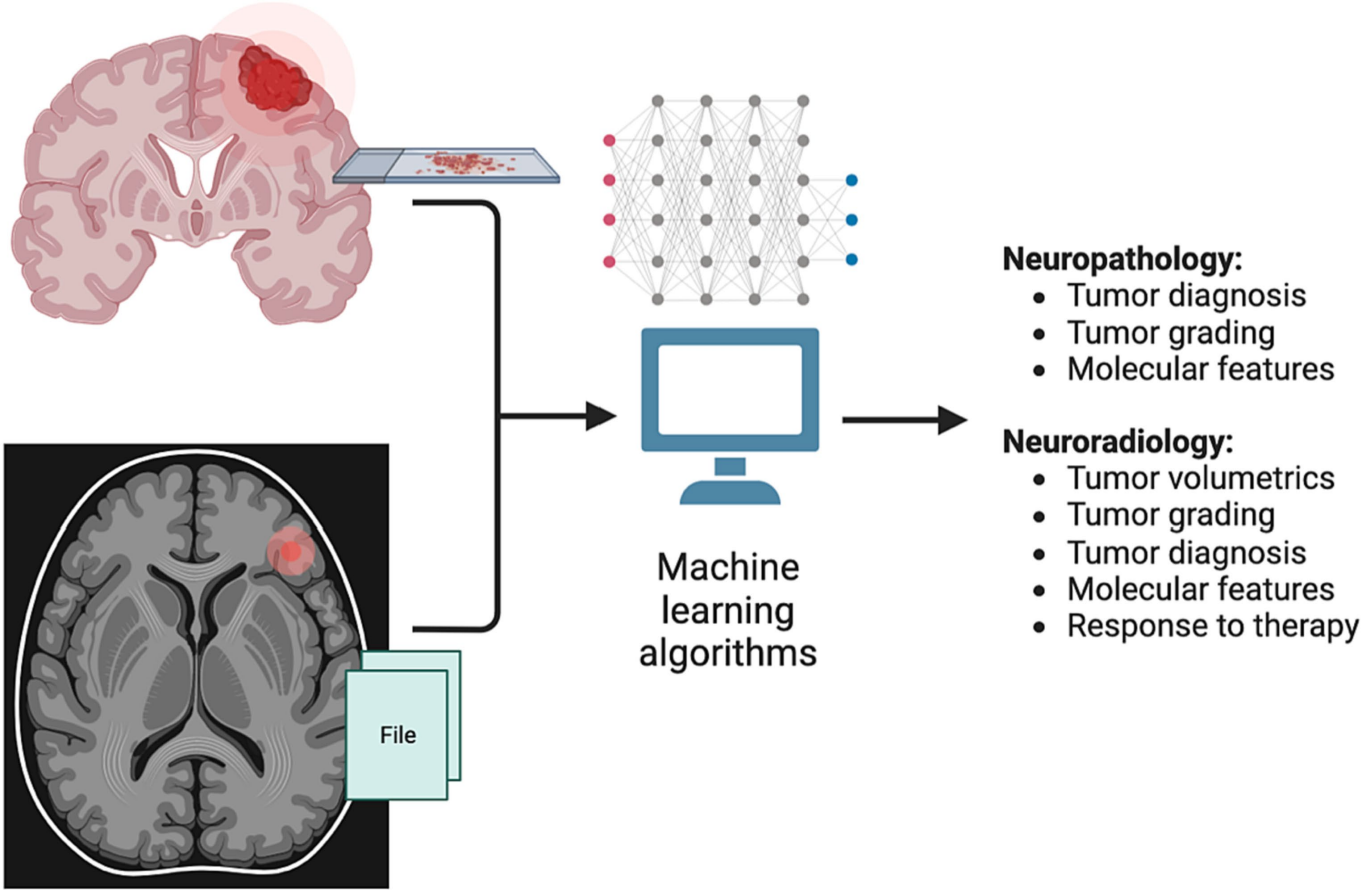
Applications of machine learning (ML) in neuro-oncology. Current research applications of ML methods in neuro-oncology have mainly introduced in the fields of neuropathology and neuro-radiology. In neuropathology, ML methods have been developed to establish a tumor diagnosis based on histopathologic and genomic features, as well as to classify tumors based on DNA methylation status. In neuroradiology, ML has been used to generate automated 3D tumor measurements (volumetrics), as well as to help predict tumor diagnosis, grading, molecular features and response to therapy.

The applications of ML to medicine, oncology, and neuro-oncology are myriad, spanning enhanced screening, diagnosis, prognosis, classification, drug discovery, precision medicine, and more (di Nunno et al., 2022). In neuro-oncology, work utilizing ML algorithms has so far predominantly been focused on neuropathology and neuroradiology applications, including tumor diagnosis and grading, prediction of molecular features, and automated assessment of tumor volume (Figure 2).

## Neuropathology applications of AI for neuro-oncology

### Histopathologic and genomic features

Histopathologic analysis has long been at the heart of diagnosis in oncology. However, it is susceptible to interobserver variability that can impede accurate diagnosis and optimized management (van den Bent, 2010). In neuro-oncology, grading of gliomas based on atypia, mitosis, microvascular proliferation and necrosis entails some degree of subjectivity that is contributory. The introduction of molecular features such as isocitrate dehydrogenase (IDH) mutation and 1p/19q co-deletion status into WHO grading of gliomas, as well as the burgeoning availability of individualized tumor genetic data, leaves AI well-positioned to assist pathologists in interpreting large and multiparametric data to establish diagnoses (see Table 2).

**Table 2:** Select studies on the application of AI/ML to neuropathology in neuro-oncology.

Authors and year	Study sample (total n)	Task	ML algorithm	Performance
Ertosun and Rubin (2015)	Gliomas grade 2–4 (44 whole tissue slides)	Glioma grade	CNN	Accuracy 96% GBM vs. LGG; 71% grade 2 vs. grade 3
Jin et al. (2021)	Glioma grade 2–4 (323 patients)	Glioma classification	CNN	Accuracy 87.5%
Pei et al. (2021)	Glioma grade 2–4 (549 patients)	Glioma grade	DNN	Accuracy 93.8% HGG vs. LGG; 74% grade 2 vs. grade 3
Hollon et al. (2023)	Diffuse glioma (373 patients)	Glioma WHO classification	CNN	93.3% accuracy
Capper et al. (2018)	Most WHO-classified CNS tumors (2801)	CNS Tumor WHO classification	Supervised ML (random forest classifier), unsupervised ML	60.4% agree with pathologist; 15.5% better subclass; 12.6% did not match pathologist but most eventually proved accurate; 11.5% unclassified

LGG, low-grade glioma; HGG, high-grade glioma; GBM, glioblastoma; WHO, world health organization; ML, machine learning; CNN, convolutional neural network; DNN, deep neural network.

The advent of high quality digitized whole slide images (WSIs) has allowed for the application of DL in histopathologic diagnosis. Broadly in oncology, DL algorithms have been used to detect metastatic breast cancer in lymph node biopsies (Litjens et al., 2016; Ehteshami Bejnordi et al., 2017), assign Gleason scores in prostate cancer biopsies (Litjens et al., 2016; Nagpal et al., 2020), and distinguish lung adenocarcinoma and squamous cell carcinoma from normal lung tissue (Coudray et al., 2018), among others, with high accuracy.

In neuro-oncology, convolutional neural networks (CNNs) trained on WSIs of gliomas have been used to render nonbiased neuropathologic diagnoses of gliomas. Ertosun et al. trained two CNNs on publicly available hematoxylin and eosin (H&E) stained histopathology images of gliomas from The Cancer Genome Atlas (TCGA). One CNN aimed to distinguish glioblastoma (GBM) vs. low-grade glioma (LGG), the other to distinguish grade 2 from grade 3 LGGs. When tested on an independent data set of glioma WSIs, the CNNs determined histopathologic grade with 96% accuracy for GBM vs. LGG and 71% accuracy for grade 2 vs. grade 3 (Ertosun and Rubin, 2015). A similar study by Truong et al. trained multiple CNNs using TCGA WSIs, with the best models achieving 73% mean accuracy in distinguishing GBM from LGG, and 53% accuracy in distinguishing grade 2 from grade 3 LGGs (Truong et al., 2020). Limitations in both included absence of IDH mutant/1p19q codeletion status of the tumors.

Jin et al. developed a platform named “AI Neuropathologist,” whereby a CNN was trained on over 79,000 H&E-stained histologic patch WSIs from 267 patients from an institutional biobank to distinguish GBM, anaplastic astrocytoma (AA), anaplastic oligodendroglioma (AO), astrocytoma (A), oligodendroglioma (O), and background glia. The CNN derived histopathologic features and classified gliomas from 56 independent patients with over seventeen thousand images into the above categories with an average patch-level accuracy of 86.5%, and patient-level accuracy of 87.5% (Jin et al., 2021). However when the tumors’ IDH/1p19q status was assessed, the numbers of patients with each genetically classified tumor subtype in the training sample were in some cases found to be relatively low (e.g., 16 “GBM with IDH mutant” and 39 “GBM with IDH-wild type”) (Komori, 2021). Im et al. used deep transfer learning to classify subtypes of gliomas from histopathologic images generated in routine clinical practice from a single institution cohort of 468 patients. Their model distinguished oligodendroglial tumors from non-oligodendroglial tumors with an accuracy of 87.3%, whereas in distinguishing glioma grade 2 vs. 3 vs. 4 the accuracy was 58% (Im et al., 2021). Pie et al. developed a deep learning-based model that fused molecular and histopathologic features to predict glioma grade. They used digital WSIs from 549 patients in the TCGA with molecular information on IDH, 1p/19q, *ATRX*, and O^6^-methylguanine-DNA methyltransferase (*MGMT*) promoter alterations. Their model achieved an accuracy of 93.8% in distinguishing high grade glioma (HGG) from LGG, and 74% distinguishing grade 2 vs. grade 3 gliomas, the latter outperforming state-of-the-art methods (Pei et al., 2021). Finally, Hollon et al. developed a DL-based method of rapid automated molecular classification of diffuse glioma from intraoperative tissue samples (Hollon et al., 2023). They trained a CNN using histologic images from 373 diffuse glioma patients, acquired by Stimulated Raman Histology (SRH) imaging. They also trained a genetic embedding model using TCGA and other public glioma genomic databases to learn labels that define molecular subgroups of diffuse gliomas. The SRH and genetic encoders were integrated to predict IDH, 1p19q, and ATRX mutations and thereby achieve molecular classification of gliomas by WHO criteria. When prospectively tested on 153 patients, the model predicted WHO glioma classification with a mean 93.3% accuracy, including IDH mutation (94.7%), 1p19q co-deletion (94.1%), and ATRX mutation (91.0%).

### Tumor classification based on DNA methylome profiling

In addition to histopathology and direct genomic alterations, DNA methylome profiling has emerged as a valuable method for classifying CNS tumors. Cancer cells undergo substantial alterations in DNA methylation patterns, which when profiled by epigenome-wide methylation assays may be used to classify tumor types with high specificity (Moran et al., 2016). Seminal work in harnessing the methylome was conducted by Capper et al., who developed a ML algorithm to classify CNS tumors based on DNA methylation profiles (Capper et al., 2018). The authors trained the algorithm with methylation data for 2,801 pre-classified samples of almost every CNS tumor type. The algorithm used supervised machine learning to recognize methylation patterns based on the known classifications, as well as unsupervised learning to search for patterns to independently assign samples into computer generated categories. In so doing the algorithm assigned the tumors to 82 distinct classes – around one-third matched known WHO tumor types; one-third represented sub-classes of WHO tumor types; notably, the remainder were classifications that did not match WHO groupings, including previously unrecognized tumor types, and those with histologic overlap but distinct methylation profiles. When prospectively tested on 1,104 new samples, the algorithm’s classification matched the pathologist’s diagnosis in 60.4% of cases; in 15.5%, the two classifications matched but the algorithm classified the tumor into a subgroup that could not be assigned by histopathology alone. In 12.6% of the cases, the algorithm’s diagnosis did not match the pathologist’s and, remarkably, further analysis (including by gene sequencing) revealed that 92.8% of these unmatched tumors were reclassified from the pathologists’ diagnosis to the algorithm’s diagnosis, including a majority that were assigned a new tumor grade. Finally, 11.5% could not be classified by the algorithm (Capper et al., 2018; Machine Learning Improves Diagnosis of CNS Cancers, 2018; Wong and Yip, 2018). Since then, multiple studies have corroborated the algorithm’s fidelity, and it has been incorporated into clinical pipelines at centers across the world (Capper et al., 2018; Jaunmuktane et al., 2019; Karimi et al., 2019; Priesterbach-Ackley et al., 2020). It has been especially useful in the classification of tumors with morphology that is heterogeneous or otherwise challenging to distinguish, including ependymomas, medulloblastomas, and diffuse glioneuronal tumors (Capper et al., 2018; Pickles et al., 2020). Its utility in guiding diagnoses for these tumors has been incorporated into the 2021 WHO guidelines for CNS tumor classification (Louis et al., 2021).

## Neuroradiology applications of AI for neuro-oncology

MRI imaging is the mainstay of diagnosis, radiographic surveillance, and assessment of treatment response in neuro-oncology. However, MRI interpretation in brain tumor patients can sometimes be challenging – treatment related changes may resemble tumor progression; histologic and molecular features that drive prognosis and guide treatment often lack readily apparent imaging correlates; and determining tumor size can pose a challenge in tumors with heterogeneous and infiltrative components. AI methods including ML, DL, and radiomics have been employed to extract from images clinically relevant information that may not be apparent visually (see Table 3). Radiomics is the process of extracting quantitative and mineable data or “features” (e.g., shape, intensity, texture) from clinical imaging. ML methods are often used to build models using these features that can predict various clinical variables. In neuro-oncology, ML/DL have been used to quantify tumor size and type, predict tumor grade, molecular features, and survival. Typically, MRI data are pre-processed and standardized, labeled/annotated by radiologists to establish ground truth for training of ML algorithms, and then may undergo augmentation, transformation and further pre-processing before being used for the training of ML/DL algorithms (Zhu et al., 2022). Often the performance of these trained algorithms is assessed on a “test” cohort of patient images not encountered in training. Technical aspects of AI in brain tumor imaging have also been reviewed elsewhere (Afridi et al., 2022; Aftab et al., 2022).

**Table 3:** Select studies on the application of AI/ML to neuroradiology in neuro-oncology.

Authors and Year	Study sample (total n)	Task	ML algorithm	Performance
Chang et al. (2019)	Newly diagnosed LGG/HGG (800), longitudinal newly diagnosed GBM (50)	tumor volumes and RANO measurements	DL	Double baseline MRIs ICCs >0.97; manual vs. automated ICC > 0.85
Kickingereder et al. (2019)	Mostly gliomas (1027)	Tumor volumes	ANN	DICE coefficients 0.91 (T1-post), 0.93 (T2/FLAIR)
Zhang et al. (2017)	HGGs (120)	IDH mutation status	Random forest	Accuracy 89%, AUC 0.92
Chang et al. (2018)	LGGs and HGGs (259)	Automated segmentation; IDH, 1p/19q, *MGMT* promoter status	CNN	Accuracy 94% (IDH), 92% (1p/19q), 83% (MGMT promoter)
Akkus et al. (2017)	LGGs (159)	1p/19q status	CNN	Accuracy 87.7%
van der Voort et al. (2019)	Presumed LGG on pre-op MRI (413)	1p/19q status	Support vector machine	AUC 0.72
Yogananda et al. (2020)	LGGs and HGGs (368)	1p/19q status	3D CNN	Accuracy 93.46%
Zhou et al. (2019)	Grade 2–4 glioma (744)	IDH and 1p/19q status	Random forest	AUC 0.92 (IDH status); accuracy 78.2% (3-group classification)
Cluceru et al. (2022)	Grade 2–4 glioma (531)	IDH and 1p/19q status	CNN	Accuracy 85.7% (overall 3-group), 95.2% (IDHwt), 88.9% (IDHmut-intact), 60.0% IDHmut-codel
Korfiatis et al. (2017)	GBM (155)	*MGMT* promoter status	CNN	Accuracy 94.9%
Chen et al. (2022)	Diffuse glioma (111)	*MGMT* promoter status	CNN	Accuracy 91%; AUC 0.90
Sun et al. (2019)	Midline glioma (100)	H3 K27 mutation status	DL	AUC 0.85
Prasanna et al. (2017)	GBM (65)	Survival	Random forest	CI = 0.70 (short term vs. long term survival)
Park et al. (2020)	GBM (216)	Survival	LASSO cox regression	CI = 0.70 (overall survival)
Beig et al. (2018)	GBM (115)	Survival	Random forest	CI = 0.83 (short term vs. long term survival)
Kim et al. (2019)	GBM (95)	Progression vs. Pseudoprogression	Hybrid ML-DL	AUC = 0.85

ICC, interclass correlation coefficient; AUC, area under the curve (referring to receiver operating characteristics curve); ANN, artificial neural network; CNN, convolutional neural network; LGG, low-grade glioma; HGG, high-grade glioma; GBM, glioblastoma; IDH, isocitrate dehydrogenase; MGMT, O^6^-methylguanine-DNA methyltransferase; RANO, response assessment in neuro-oncology; CI, concordance index. *n* reflects training and validation cohorts where applicable.

### Tumor volumetrics

Reliably delineating tumor size and burden on structural MRI brain is necessary to longitudinally assess tumor progression and response to treatment, and is thus critical to both effective clinical care and the assessment of response in clinical trials. However, identifying tumor boundaries manually on MRI per the RANO (Response Assessment in Neuro-Oncology) criteria, which involve quantitative 2D measurements of contrast-enhancing and FLAIR hyperintense lesions, can be challenging for infiltrative tumors like high grade gliomas, and is associated with high interrater variability (Vos et al., 2003; Pope and Hessel, 2011; Ford et al., 2016).

Chang et al. developed a deep learning algorithm to automatically segment T2/FLAIR and T1-post contrast MRI images of adult gliomas to quantify both 2D RANO measurements as well as 3D tumor volumes (Chang et al., 2019). They used MRIs from 800 patients with newly diagnosed LGG and HGGs, and over 700 post-op longitudinal MRIs from 50 patients with newly diagnosed GBMs. Their automated tumor quantification was reproducible in double baseline MRIs (interclass correlation coefficients, or ICCs, > 0.97), with high agreement between manual and automated tumor volumes (ICCs >0.91), and between manually and automatically derived longitudinal changes in tumor burden (ICCs >0.85). Though their automated RANO measurements were reproducible and internally consistent, they were often larger than manual RANO measurements. Taken together with inconsistency of RANO measurements found between the two human raters, these findings suggested that the automated measurements may be more accurate (detecting longer diameters than can be visualized by eye), and more precise. Peng et al. used a similar deep-learning approach to develop an algorithm that determines two dimensional measurements and three-dimensional volume in pediatric high grade gliomas, medulloblastomas, and other leptomeningeal-seeding tumors, with high repeatability and agreement with human raters (Peng et al., 2022).

3D volumetric measurements are not routinely used in response assessment for gliomas, in part due to the labor intensive, time-consuming, and variable nature of segmentation approaches, though they are likely more reliable and accurate than 2D measurements (Sorensen et al., 2001, 2008). Kickingereder et al. trained a DL-based CNN to carry out automated tumor segmentation on MRI data from 455 patients with brain tumors (mostly gliomas), and tested it on over two thousand MRIs from over 500 patients. The algorithm demonstrated high precision with Dice coefficients of 0.91 (T1 post-contrast) and 0.93 (T2/FLAIR) (Barash and Klang, 2019; Kickingereder et al., 2019).

Although a recent evaluation of the RANO criteria suggests that analysis of FLAIR data (when performed by humans) does not add additional information in terms of predicting survival (Youssef et al., 2023), analysis of FLAIR sequences with automated algorithms such as developed by Chang et al. and Kickingereder et al. may enable the incorporation of volumetric assessment of gliomas into research assessments and potentially clinical practice.

### Prediction of molecular features

As signified by their growing prominence in the WHO classification of central nervous system tumors in 2016 and 2021, molecular features of CNS tumors are increasingly informing diagnosis, prognosis, and management (Louis et al., 2021; Gritsch et al., 2022). For instance, the presence of an IDH mutation in adult gliomas precludes a diagnosis of glioblastoma, WHO grade 4, regardless of histologic grade given its favorable prognosis compared to IDH wildtype tumors. IDH mutant gliomas with 1p/19q co-deletion are classified as oligodendrogliomas (WHO grade 2–3), and those without 1p/19q co-deletion are classified as astrocytomas (WHO grade 2–4). This highlights the importance of molecular testing, which can be time consuming and requires adequate surgical tissue for histopathologic and genetic analysis, in routine clinical practice. Noninvasive determination of a tumor’s molecular features via imaging would be valuable not only in cases of inoperable tumors or insufficient surgical samples, but also in all cases to guide early diagnosis or early pre-surgical enrollment into clinical trials (Gonzalez Castro et al., 2023).

Certain qualitative radiographic correlates of molecular features have previously been recognized. For instance, IDH wildtype LGGs have poorer definition of non-enhancing margin and more multifocal distributions than IDH mutant LGGs; IDH wildtype tumors have larger percentage core enhancing component; and the “T2-FLAIR mismatch” sign can identify IDH-mutant 1p19q-intact gliomas with good specificity (Ellingson, 2015; Patel et al., 2017; Park et al., 2018; Lasocki et al., 2021; Miller et al., 2023). IDH mutant gliomas have been shown to have higher ADC and lower relative cerebral blood volume (rCBV) values, though these findings have wide ranging sensitivities (56 to 100%) and specificities (51–100%) depending on the study (Xing et al., 2017; Suh et al., 2019). 2-HG MR spectroscopy has better sensitivity in detecting IDH mutant status, in one analysis sensitivity 96% and specificity 85% (Suh et al., 2019). Additionally, 1p/19q co-deleted tumors are associated with indistinct tumor borders, frontal tumor location, heterogeneous T2 signal intensity, and cortical/subcortical tumor infiltration (Smits and van den Bent, 2017).

ML algorithms developed to predict molecular features based on imaging data offer the promise of automated recognition of these and other features, and have the advantage of being independent of operator experience, more accessible, and more amenable to training on improved data sets. Here we highlight some salient studies among the numerous studies that have employed ML approaches to predict molecular features including IDH mutation, 1p/19q codeletion, *MGMT* promoter methylation status, and other relevant features.

Zhang et al. developed a ML-based model using a random forest classifier to predict IDH mutation status based on patient age and pre-operative MRIs of 90 patients with HGGs. Using T1, T2/FLAIR, and ADC sequences, the model achieved accuracy of 89% (AUC 0.9231) in the validation cohort of 30 HGGs (Zhang et al., 2017). Imaging features contributing the most to IDH genotyping were patient age and MRI parametric intensity, texture and shape features. In a similar study, Chang et al. trained a CNN to predict IDH mutation status from pre-operative MRIs of patients with grade II-IV gliomas, and accuracies improved from 85.7 to 89.1% with incorporation of patient age into the predictive model (Chang et al., 2018). These models required manual tumor segmentation which limits clinical viability, but automated segmentation approaches (as described in the previous section) may help overcome this. A meta-analysis of 9 studies by Zhao et al. employing ML to radiographically predict IDH mutations in gliomas found pooled sensitivity and specificity of 87 and 88%, respectively, in the training set, and 87 and 90%, respectively, in the validation set (Zhao et al., 2020). Finally, Chang, P et al. used MRI data from 259 patients with low or high grade gliomas from The Cancer Imaging Archive (TCIA) to train a CNN to predict IDH mutation, 1p/19q codeletion, and *MGMT* promoter methylation status simultaneously and using an automated segmentation tool (Chang et al., 2018). They achieved a high accuracy of 94% in IDH mutation status, while accuracies for 1p19q codeletion and *MGMT* promoter methylation were 92 and 83%, respectively.

Codeletion of the 1p/19q chromosome arms in IDH mutant gliomas is characteristic of oligodendroglioma and associated with increased survival and better response to treatment, and is another important part of glioma classification by WHO criteria (Taal et al., 2015; Louis et al., 2021). Fellah et al. used multivariate random forest models to retrospectively predict 1p/19q codeletion status from conventional MRI (cMRI) sequences (T1- and T2-weighted sequences) and from diffusion-weighted imaging (DWI), perfusion-weighted imaging (PWI), and MRI spectroscopy (MRS). Their model had misclassification rate of 48% and established that inclusion of DWI, PWI, and MRS did not help improve the prediction of 1p/19q codeletion relative to cMRI sequences alone (Fellah et al., 2013). Akkus et al. used 159 preoperative cMRIs of LGGs to train and test a CNN to predict 1p/19q codeletion status, and achieved an accuracy of 87.7% (Akkus et al., 2017). Van der woort et al. trained a support vector machine (SVM) algorithm on cMRI images of 284 patients who had undergone biopsy or resection for presumed LGG (rather than histologically confirmed LGG, so as to reflect a more clinically relevant population). Their model, which also incorporated age and sex data, predicted 1p/19q codeletion in 129 patients from an external test cohort from the TCIA, with AUC of 0.72. The authors compared this to predictions by clinical experts, who achieved AUCs of 0.52 (two neurosurgeons) and 0.81 (two neuroradiologists) albeit with wide variability among the clinical experts (AUC 0.45–0.83) (van der Voort et al., 2019). Finally, Yogananda et al. used only T2-weighted MRI sequences from a cohort of 368 patients from the TCIA/TCGA with low and high-grade gliomas, divided into training, validation and testing sets, to predict 1p/19q-codeletion. Their 3D CNN achieved an accuracy of 93.46% (Yogananda et al., 2020). Their exclusive use of T2-weighted images, as well as of automated tumor segmentation, signified a step forward in terms of potential implementation in a clinical setting.

Given the need to identify multiple molecular alterations simultaneously (e.g., IDH and 1p/19q codeletion status) for accurate classification of gliomas, some investigators have worked to develop models for simultaneous classification into one of 3 groups: IDH wild type (IDHwt), IDH mutant and 1p/19q-codeleted (IDHmut-codel), and IDH mutant and 1p/19q non-codeleted (IDHmut-non-codel). Matsui et al. used multi-modal MRI ^11^C,-methionine PET, and CT images as well as age/gender data from 217 LGG patients to develop a DL model to predict glioma classification, achieving 68.7% accuracy in the test dataset. They noted lower accuracies with only MRI, MRI and PET, and MRI and CT, and reasoned that ^11^C-methionine-PET increased yield for oligodendrogliomas and IDH wild type astrocytomas, while CT increased yield for oligodendrogliomas by detecting calcification (Matsui et al., 2020). Zhou et al. trained a random forest algorithm on preoperative cMRI in 538 patients with grade 2–4 gliomas from three different institutions. Integrating patient age, they developed two models to sequentially detect IDH mutation status, then 1p/19q status among the IDH mutants. When tested on an external validation cohort from the TCIA of 206 patients with glioma, their model achieved AUC of 0.919 for IDH mutation, and an overall accuracy for glioma classification of 78.2% (Zhou et al., 2019). The authors suggest that a larger sample size may enhance 1p/19q codeletion status prediction in this model. Finally, Cluceru et al. trained a CNN to identify IDH mutation and 1p/19q co-deletion in pre-operative MRIs of newly diagnosed grade 2–4 gliomas, using a cohort of 384 patients from a single institution and 147 patients from the TCGA dataset (Cluceru et al., 2022). They trained multiple CNN classifiers, including using a sequential model (predicting IDH mutation first, then 1p19q codeletion) and a simultaneous 3-group model; they also trained CNNs with or without DWI sequences in addition to cMRI sequences. They found that their best classifier was a 3-group CNN that included DWI as input, predicted molecular features with an overall test accuracy of 85.7%, and correctly classified 95.2% IDHwt, 88.9% IDHmut-intact, and 60.0% IDHmut-codel gliomas. The authors suggested that incorporating susceptibility-weighted imaging (SWI) and rCBV sequences into future algorithms may improve diagnostic accuracy in IDHmut-codel gliomas.

Methylation of the *MGMT* promoter in gliomas predicts longer survival and better response to alkylating chemotherapy agents such as temozolomide, and is thus a clinically vital molecular feature to determine (Stupp et al., 2009). Radiographically, gliomas with *MGMT* promoter methylation have been associated with less vasogenic edema, higher ADC values, and lower cerebral blood flow and blood volume on MR PWI, relative to unmethylated tumors according to a meta-analysis of relevant studies (Suh et al., 2019). Several studies have endeavored to noninvasively assess *MGMT* promoter methylation status via MRI using ML and DL methods.

Li et al. used a cohort of 193 patients with newly diagnosed GBM to build a ML-based random forest classifier for prediction of *MGMT* promoter methylation status in pre-operative cMRIs. Their model selected six features including location, geometry, intensity and texture features; it predicted *MGMT* promoter methylation status with 80% accuracy (AUC 0.88), and the addition of clinical features did not lead to an improvement of this result (Li et al., 2018). Crisi et al. used MR PWI in a cohort of 59 patients with GBM to identify 14 quantitative radiomic features that were used to build a DL model to classify *MGMT* promoter methylation status into three groups: unmethylated (<10% methylated), intermediate-methylated (10–30% methylated), and methylated (>29% methylated). Their model classified *MGMT* promoter methylation status into these three groups with AUC 0.84, sensitivity 75% and specificity 85% (Crisi and Filice, 2020). This lends support to MR PWI as a potential biomarker for *MGMT* promoter methylation status using ML/DL classifiers. Korfiatis et al. used T2 MRI images from 155 patients with newly diagnosed GBM to train and test three different residual CNNs to predict *MGMT* promoter methylation status in each image slice. Their best performing CNN had 50 layers, and predicted MGMT status (methylated, unmethylated, or no tumor) with 94.90% accuracy in the test set. Notably their model eliminated the need for a manual tumor segmentation step (Korfiatis et al., 2017). Chen et al. built a DL model to assess the predictive value of cMRI and ADC sequences in 111 patients using two regions of interest (ROIs), tumor core and tumor whole (the latter including tumor edema). They found highest predictive value in the tumor core ROI using T1-post contrast combined with ADC sequences, with 91% accuracy and AUC 0.90 (Chen et al., 2022).

A review and meta-analysis of ML-based prediction of molecular features in glioma using MRI by Jian et al. examined 44 studies and found a pooled sensitivity and specificity for IDH mutation of 0.83 and 0.85, respectively. Pooled sensitivities and specificities for 1p/19q codeletion and *MGMT* promoter methylation ranged between 0.76 and 0.83. Of the 44, 7 studies utilized DL, while most used ML-based random forest or SVM classifiers (Jian et al., 2021). Another review and meta-analysis by Bhandari et al. on using MRI radiomics to predict IDH and 1p/19q status in LGGs examined 14 studies. They found that for IDH mutation status prediction, conventional radiomics combined with DL based CNN derived features was the most accurate approach, with 94.4% sensitivity and 86.7% specificity. In contrast, conventional texture-based radiomics performed best in predicting 1p/19q codeletion status, with 90% sensitivity and 96% specificity (Bhandari et al., 2021). These results should be interpreted cautiously, as there was a high degree of heterogeneity among the studies reviewed, with varying radiomic pipelines many of which required manual tumor segmentation, making direct comparisons challenging.

In diffuse midline gliomas, H3 K27 mutation is commonly observed in both pediatric and adult patients, and in pediatric patients portends decreased overall survival regardless of tumor location or histopathological grade (Karremann et al., 2018; Kleinschmidt-DeMasters and Mulcahy Levy, 2018; Ebrahimi et al., 2019). As many of these tumors are located in the brainstem, surgical intervention, including biopsy, can be morbid and is sometimes foregone, increasing the utility of accurate non-invasive H3 K27 mutation status prediction. Su et al. developed deep learning models to predict H3 K27 mutation using only T2 weighted MRI sequences in a cohort of 100 patients with midline gliomas, including 40 mutant and 60 wild type tumors, with three quarters of the cohort reserved for a training set and one quarter for testing. Of ten generated prediction models, accuracies ranged 60 to 84% in the testing cohort, and the best model had a AUC of 0.85 in the test cohort. Larger sample sizes, may help further refine the accuracy of this approach.

### Prognostic models

Discussing prognosis is of major importance at the time of brain tumor diagnosis, especially for GBM where the median survival is approximately 16–18 months despite completion of standard-of-care therapy (Wen et al., 2020). Risk factors for poor survival in GBMs include older age and lower Karnofsky Performance Scale (KPS) scores at time of diagnosis, surgery without adjuvant chemoradiation, and absence of *MGMT* promoter methylation (Krex et al., 2007; Thumma et al., 2012). Radiographic MRI features have also been associated with worse overall survival including degree of necrosis and contrast enhancement, multifocality, peritumor edema and higher rCBV (Hammoud et al., 1996; Lacroix et al., 2001; Pope et al., 2005; Jain et al., 2014).

ML and DL-based algorithms have been developed and evaluated to predict survival using a combination of radiographic and clinical features. Sun et al. used a 3D CNN for automated segmentation of cMRI images from 210 HGG and 75 LGG patients, and then used a ML-based random forest classifier to extract radiomics features and predict overall survival. They classified 66 gliomas in a validation cohort into short-term (<10 months), mid-term (10–15 months) and long-term (>15 months) survivors with a modest (61%) accuracy (Sun et al., 2019). Prasanna et al. used cMRI sequences from 65 patients with GBM from the TCIA, manually segmented into enhancing, peritumoral brain zone, and tumor necrosis regions; they extracted 402 radiomics features and used a random forest classifier to isolate features most predictive of short-term (< 7 months) vs. long term (>18 months) survival. They found that peritumoral radiomic features combined with multiparametric MRI sequences performed best at predicting long- vs. short-term survival with a concordance index (CI) of 0.70 (as opposed to combining tumor necrosis features with specific T1 or T2 sequences). When combined with clinical features the model’s highest predictive accuracy was achieved at a CI of 0.735 (Prasanna et al., 2017). Lao et al. developed a DL-based model using cMRI combined with clinical data (age and KPS) from 112 patients with GBM from TCIA and institutional cohorts, to predict overall survival with a similar CI of 0.710 (Lao et al., 2017).

Nie et al. used T1 MRI, resting state functional MRI (rs-fMRI), and diffusion tensor imaging (DTI) from 68 HGG patients, and develop a 3D CNN to extract predictive radiomics features. These were combined with clinical features including age, gender, tumor location/size, and WHO grade, and incorporated into a SVM model to predict short vs. long overall survival time, defined as less than, or greater than 650 days, respectively, with 88% accuracy on a 25 patient validation cohort (Nie et al., 2019). Limitations of this study include the its small sample sizes, as well as a binary cutoff of 650 days defining short- vs. long-term survival. Park et al. extracted radiomics features from MRI DWI and PWI in addition to cMRI from 158 patients with newly diagnosed GBM, and combined these with clinical features including age, gender, KPS, *MGMT* promoter methylation status, and extent of surgical resection to develop a ML-based predictive model for survival. On a test set of 58 patients the model predicted OS with a CI of 0.70, performing better than the authors’ models that used radiomics features or clinical predictors alone (Park et al., 2020).

As tumor hypoxia is considered an important molecular mechanism driving treatment resistance and poor prognosis, Beig et al. aimed to study radiomics features that predict tumor hypoxia, and utilized these to develop a predictive model for survival in GBM. Radiomics features extracted from cMRI of 115 subjects from the TCIA, coupled with RNA seq data from 21 genes implicated in GBM hypoxia, were used to generate a hypoxia enrichment score (HES). A random forest classifier was then used to stratify patients into short-term (OS <7 months), mid-term (OS 7–16 months) and long-term (OS >16 months) survival based on radiomic markers of hypoxia and clinical features (age, gender, KPS). On a validation subset, the model was able to predict a statistically significant separation between the Kaplan–Meier curves of short-term and long-term survivors, with a CI of 0.83 (Beig et al., 2018). In addition to predicting survival, non-invasive assessment of tumor hypoxia may guide selection of patients for clinical trials or management with anti-angiogenic therapy (Rahman et al., 2010). Future studies on prognostication may benefit from greater incorporation of molecular features, including IDH mutation, *MGMT* promoter methylation and 1p/19q codeletion status into predictive models.

### Differentiating progression from treatment-related radiographic changes (PseudoProgression)

Assessment of true progression (TP) of brain tumors (particularly HGG) on surveillance MRI often presents a significant clinical and radiologic challenge as true progression can appear radiographically similar to pseudoprogression (PsP), i.e., radiation treatment-related inflammatory changes most common 3–6 months after completing radiotherapy (Ellingson et al., 2017). Distinguishing TP from PsP is vital in guiding management and enrollment in (or withdrawal from) clinical trials. In practice, while pathological diagnosis is often considered gold standard to distinguish the two, serial MRI is often used for practical reasons as treatement-related changes regress over time (Youssef et al., 2023). However, this approach can lead to diagnostic delay. Moreover, TP and PsP may co-exist. Studies have suggested that recurrent tumors have lower ADC values than radiation necrosis on DWI sequences, and higher rCBV on PWI can predict PsP with 81.5% sensitivity and 77.8% specificity (Kong et al., 2011; Chu et al., 2013). Approaches utilizing radiomics, ML and DL have ventured to make this distinction noninvasively in the hopes of improving diagnostic fidelity.

Kim et al. studied cMRI, ADC and CBV sequences in 61 patients with GBMs who had undergone resection and standard concurrent chemoradiation therapy (CCRT), and had developed new contrast enhancing lesions within 12 weeks of completion of the latter. Ground truth of TP vs. PsP was based mostly on subsequent serial MRIs, though 8 cases were confirmed with pathology. They extracted radiomics features from the contrast-enhancing portion of the MRIs and used a ML-based classifier to develop a model to distinguish TP vs. PsP. Their multiparametric model (incorporating cMRI, ADC, CBV) performed the best with AUC 0.85 on an external validation cohort of 34 patients (Kim et al., 2019). Jang et al. used a similar cohort of 59 GBM patients to train a hybrid ML-DL model with CNN-LSTM (long short-term memory) on T1 pre- and post-contrast MRI, as well as clinical and molecular features, and were able to distinguish TP from PsP with AUC of 0.83 on an external validation set of 19 patients (Jang et al., 2018). Pathologic confirmation was available for 20 TP and 3 PsP cases. In a similar study employing data from 124 GBM patients with new enhancing lesion after resection and CCRT, Moassefi et al. trained a CNN that achieved AUC 0.75 in distinguishing TP from PsP, with all ground truth determination of TP vs. PSP based on serial imaging (Moassefi et al., 2022).

## Discussion

The practice of neuro-oncology is developing at an ever-faster pace, propelled by advances in our understanding of brain tumor biology and technical innovations in allied fields such as neuropathology and neuroradiology. In parallel, advances in AI methods hold increasing promise to optimize workflows in many aspects of neuro-oncology care, as well as to generate new insights regarding tumor biology and therapeutic mechanisms. In neuropathology, to date, AI algorithms have been applied to WSI data to resolve histopathologic features, aiding brain tumor diagnosis and grading. In addition, ML is increasingly being applied to tumor classification on the basis of DNA methylome profiling. In neuroradiology, AI algorithms have been applied to the problem of tumor measurement (volumetrics), to the prediction of grade, molecular features and diagnosis, as well as to the discrimination between progression and treatment-related changes, and the determination of prognosis. As highlighted above, the accuracy of the output of many of these analyses depends on the complexity and diversity of the training datasets, and the AI methods applied to tackle each problem. There is clearly room for improvement, and this is expected through collaboration across centers (leading to more extensive and diverse datasets) and improvements in computational methods and hardware.

Witnessing the current progress, a natural question is if these algorithms will one day come to replace the work of neuropathologists and neuroradiologists in neuro-oncology practice. Although the roles of neuropathologists and neuroradiologists will evolve, we do not expect these specialists to come “out of the loop,” as their expertise is irreplaceable, particularly when it comes to diagnosing and evaluating difficult cases. AI will not replace neuropathology or neuroradiology but rather expedite and enhance their workflows. With advances in large language models (LLM; e.g., ChatGPT), which are able to address complex queries with increasing accuracy (Haupt and Marks, 2023), the relevance of the clinical neuro-oncologist also comes into question. Here again, we think that AI will not replace but rather support the role of neuro-oncologists, putting the latest clinical evidence and treatment algorithms at their fingertips, systematizing part of their role but unable to replace the physical touch that enables patient assessment and the development of a relationship that helps guide patients through difficult decisions.

As the field of AI continues to develop and progressively integrate into research and clinical practice, we need to remain aware of the limitations of each method/algorithm, particularly since their underpinnings are often not clearly explained and, more importantly, are difficult to assess by end users. Guidelines for evaluating, validating and approving AI systems for their use in medicine in general, and neuro-oncology specifically, will be fundamental to the safe introduction of these methods into practice. Elements to consider when evaluating novel AI tools include, (1) the characteristics of training datasets (data types and standards, diversity of dataset elements, size of dataset, accuracy of data annotations, if relevant), (2) the specifics of the algorithms involved, (3) the characteristics of the validation dataset (including metrics that are consistent with those of the training dataset), and (4), the performance of the system at the moment of its release as well as over time, including specific warnings regarding blindspots of classification or systematic errors regarding output for specific inputs. Related to this last point, it is important to note that AI systems have the potential to perpetuate clinical and social biases (Larrazabal et al., 2020; Seyyed-Kalantari et al., 2021).

In the end, how AI will continue to integrate into the practice of neuro-oncology remains to be determined. We hope to have updated neuro-oncology clinicians and researchers on current advances in the field of AI to help them inform how to incorporate AI tools into their practice. In the words of the Nobel Prize winning physicist, Dennis Gabor, “the future cannot be predicted, but futures can be invented (Gabor, 1964).”

## Author contributions

VN performed the literature review and drafted the manuscript. LG conceived the project, performed the literature review, and drafted the manuscript. All authors contributed to the article and approved the submitted version.
